# Study on the mechanism of regulating the hypothalamic cortical hormone releasing hormone/corticotropin releasing hormone type I receptor pathway by vibro-annular abdominal massage under the brain–intestine interaction in the treatment of insomnia

**DOI:** 10.1097/MD.0000000000025854

**Published:** 2021-05-14

**Authors:** Ye Zhang, Deyu Cong, Peng Liu, XiaoYu Zhi, Congcong Shi, Jiajun Zhao, Hongshi Zhang

**Affiliations:** a Acupuncture and Massage College of Changchun University of Chinese Medicine; b Affiliated Hospital of Changchun University of Chinese Medicine; c Nursing College of Changchun University of Chinese Medicine, Changchun, China.

**Keywords:** 1H nuclear magnetic resonance, 5-HT, brain and intestine interaction, insomnia

## Abstract

**Background:**

Insomnia is a common disease associated with different nervous system stress response and endocrine disorders. It has been reported previously that abdominal vibration and ring massage therapy can significantly improve the symptoms of insomnia patients, enhance the activity of neurons. In addition, functional MRI (resting state brain functional magnetic resonance imaging [Rs_fMRI]) of the resting state brain test has proved that the functional connection between hypothalamus and parahippocampal gyrus could be significantly enhanced after abdominal massage treatment. It has been confirmed that there is possible involvement of brain–gut interaction effect in the treatment of insomnia, but there is a lack of research to elucidate the possible mechanisms of brain–gut interaction in the treatment of insomnia. The purpose of this study is to investigate the relationship between the hypothalamus and intestinal interaction in the treatment of insomnia by abdominal massage.

**Methods and design:**

A single blind randomized controlled trial will be conducted. Sixty chronic insomnia volunteers and 30 healthy volunteers will be recruited for this study. Sixty insomnia volunteers will be randomly divided into a drug group and a massage group, and 30 healthy volunteers will be assigned to the healthy group. The manipulation of the treatment group will be mainly carried out through abdominal rubbing and vibration massage, once a day, 30 min/time, 5 days for a course of treatment, and a total of 4 intervention courses will be carried out. Patients in the drug group will be given orally spleen-invigorating bolus, twice a day, 1 pill in the morning and 1 pill in the evening. The course of treatment will be carried for 5 days, and a total of 4 courses of treatment will be administered.

The massage group will be compared with the healthy group and the drug group by Pittsburgh Sleep Index scale (PSQI), Hyperarousal scale (HAS), Hamilton Depression scale (HAMD), Fatigue scale-14 (FS-14), and Wechsler Adult Memory scale (WAIS) scales using to observe the sleep quality. Rs-fMRI will be used to observe various BOLD signals in the brain and compare the values of Reho, fALFF, and FC. MRS technology will be used to observe the contents of GABA and 5-HT in the hypothalamus. Additionally, the contents of cortical hormone releasing hormone (CRH), adrenocorticotropic hormone (ACTH), COR, GABA, NE, PGE2, and 5-HT in the serum will be also detected. The serum of each group will be taken for 1H nuclear magnetic resonance (^1^HNMR) metabolomics study to analyze the various common metabolites, differential metabolites, potential metabolic biomarkers, and metabolic pathways among the 3 groups. Finally, in combination with the brain functional imaging and brain spectrum, the potential mechanism of abdominal vibration and ring massage will be discussed.

**Discussion:**

The results of this study will be used to possibly elaborate the various mechanisms of brain and intestine interaction in the treatment of insomnia by employing abdomen ring rubbing.

**Advantages and limitations of proposed study**

- The study will comprise of a safe and reliable clinical trial, which will only perform abdominal massage in patients with insomnia with almost no risk as well as high comfort level, and will thereafter investigate the possible therapeutic effects of abdominal massage on patients with insomnia.
- After treatment, the volunteers and patients will be assessed by PSQI, FS-14, as well as heart rate variability (HRV), and RS_fMRI, MRS, and serum metabolomics will be detected. The techniques used will be safe, harmless, and will be able to explain the potential difference between insomniacs and healthy volunteers.
- The basic advantages of this study will be its safety, reliability, practicability, and high comfort level. In this clinical trial, patients will be treated in a standard way, and the rights and interests of the subjects will be fully guaranteed. The limitation is the lack of double-blind control, and the drug group uses the recommended drug in the TCM guidelines.

## 1 Introduction

Insomnia is the insufficient sleep time and quality and could significantly affect the subjective experience of the daytime activities. Additionally, with the rapid increase in the pace of life, as a result of significant work pressure, the incidences of insomnia, has gradually increased and patients may have trouble falling asleep and could exhibit sleep disorders, mood disorders, fatigue, irritability, causing a decrease in the work and learning efficiency. These changes can lead to serious depression, and could cause chronic heart cerebrovascular disease and neural system diseases. From the perspective of etiology, insomnia can be divided into primary insomnia and secondary insomnia, and according to the inheritance it can be divided into acute insomnia (<4 weeks) and subacute insomnia (>4 weeks and <6 months) as well as chronic insomnia (>6 months). At present, one of the major treatment options is the drug intervention to treat insomnia and the other is cognitive behavioral therapy. However, traditional Chinese medicine (TCM), complementary and alternative therapies are still not recommended in European Guideline for Diagnosis and Treatment of insomnia due to the poor quality of medical evidences to substantiate their effectiveness.^[3]^ So the investigation of potential mechanisms by which massage treatment could insomnia and the understanding of various nerve, endocrine system, and immune disorders to clarify the main direction of the pathogenesis of the insomnia is important. In the nerve—endocrine system by the hypothalamus-pituitary-adrenal axis (HPA) mediated hormonal balance. This could be of great significance in the management of insomnia.^[4]^

Abdominal vibration and ring massage therapy tradition medicine of Changbai mountain use meridians to adjust the internal organs means in the genre,^[5]^ based on the visceral massage, thereby combining modern brain–gut interaction theory. This could gradually lead to forming a “improve the brain by treating the intestines” featuring technique, safe and reliable clinical operation with obvious therapeutic impact, and no severe side effects. Moreover, bead on the results of our previous national natural fund project entitled “the abdominal massage therapy type glove 2 virtual research on the brain–gut interaction mechanism of primary insomnia (no. 81574094)” it was confirmed that the abdominal vibration and ring massage could significantly improve the symptoms of patients with insomnia, and thereby enhance neuronal activities. It could also improve the substance P in the ghrelin, neuropeptide Y, and enkephalin disorder state of matter.^[6]^ The findings of resting state brain functional magnetic resonance imaging (Rs_fMRI) test also proved that the functional connection between hypothalamus and parahippocamal gyrus was significantly enhanced after abdominal massage treatment.^[7]^ It has been proved that there possibly exists a brain–gut interaction in the treatment of insomnia. However, due to various limitations in the clinical diagnosis, treatment standards, and medical ethics, there is still a lack of research on the specific mechanism of brain–intestinal interaction in the treatment of insomnia by abdominal massage.

Hypothalamus-pituitary-adrenal gland (HPA) is an important mechanism for coordinating the nervous system and endocrine system. It participates in the stress response of the nerve center, and also controls the regulation of gastrointestinal smooth muscle contraction and mucosal transport.^[8]^ The changes in the gastrointestinal motility, mucosal secretion, and intestinal permeability will have a significant impact on the microbiota in the gastrointestinal tract. HPA axis system consisting of the hypothalamus paraventricular nucleus of the hypothalamus (PVN) is one of the important regulating center brain–gut interaction mechanism. The hypothalamus paraventricular nucleus can secrete adrenal cortical hormone releasing hormone (CRH),^[9]^ that can stimulate the activation of HPA activation, CRH can then bind to corticotropin releasing hormone type I receptor (CRHR1) through its receptor binding reaction, thereby promoting the pituitary to secrete adrenocorticotropic hormone (ACTH). This hormone then circulates throughout the body and can stimulate the adrenal glands, leading to the secretion of cortisol (CRO) from the adrenal cortex. CRO has been reported to be an important medium between HPA axis and the human body endocrine and closely associated with sleep. CRO is a glucocorticoid, which is widely used clinically as an effective anti-inflammatory and immunosuppressive agent.^[8]^ Moreover, a number of reports have found that sleeping <5 hours in cases may be associated with CRO,^[10]^ as in the beginning of sleep HPA axis is restrained, and CRO secretion can be reduced transiently. However, generally in the middle of the night when CRO level reaches its lowest concentration, it can lead to a gradual increase in the activity of HPA axis after the start of the sleep and this increase has been reported to peak around 9 o ’clock in the morning.^[11]^ Thus, the concentration of CRO and ACTH can be effectively detected in the blood samples of insomniacs, and it has been found to be more obvious in the afternoon, evening, and early morning. Additionally, both ACTH and CRO could potentially serve as an important factor in research on insomnia and HPA axis, but the available research data of patients with insomnia and HPA axis, only involves the role of ACTH and CRO, but has limited information on the pathway upstream substances. As the initiating factor of the hypothalamus, CRH is a key substance of the HPA axis. It is a key substance that causes insomnia after the human body is exposed to stress. At present, relevant research on the HPA axis of insomnia patients should primarily focus on the role of various upstream substances regulating this pathway.

The proposed clinical trial will determine the possible effects of abdominal ring-rubbing method on sleep quality of insomnia patients by using neuropsychology, resting state brain functional MRI (RS-fMRI), and metabonomics, and will clarify the role of the brain–gut interaction mechanism of abdominal ring-rubbing method in improving the sleep quality.

## 2 Methods and design

### 2.1 Objectives

Study objectives: to determine the potential efficacy of treatment of insomnia ring vibrating abdominal kneading method. To decipher the various neural mechanisms underlying vibrating abdominal ring rubbing method for treating insomnia.

### 2.2 Setting

The patients will be recruited from the Department of Zang-fu Massage, Affiliated Hospital of Changchun University of Chinese Medicine. Additionally, for participating in the trial, the patients will be screened strictly according to the diagnostic criteria, inclusion criteria, and exclusion criteria, and the requirements for the sample size estimation will be based on the clinical calculation formula employed for small sample size. The sample size (n = 16.76) will be calculated, that is, the therapeutic effect could be judged by the sample size of >16.67 people in each group. In this study, therefore 30 people will be observed in each group, which met the sample size estimation criteria.

The trial researcher will be able to record the subject's basic information (such as date of birth, sex) and answer patient trial screening questions. After the various patients have completed screening and enrolled, the trial study serial number, that is, the patient's unique identification number (SIN), will be obtained according to the enrollment time and order of enrollment, until the total number of observations (30 cases) was completed. Thirty healthy volunteers and 30 patients will be recruited for the study. After clinical evaluation and blood sampling, abdominal massage therapy will be adopted.

### 2.3 Participants and recruitment

The recruitment posters will be displayed in the hospitals and forward recruitment information will be posted on WeChat to recruit the potential participants for this study. The participants will be explained in detail about the potential benefits and services to be provided and the risks associated with participating in the study, including adverse clinical outcomes that may be associated with abdominal massage therapy. A detailed reading and an informed consent will be signed by each participant prior to the participation. On the consent form, participants will also be asked whether they provide consent to use their data even if they opt out of the trial. The participants will also be required to obtain permission for the research team to share relevant information with staff from the participating hospitals or regulatory agencies. The participants will receive 4 different courses of abdominal massage therapy. In addition, 30 healthy age- and sex-matched, volunteers with good sleep pattern will be recruited as the healthy controls. The personal information of all the participants will be kept confidential by the researchers and used only for this study.

**Figure 1:**
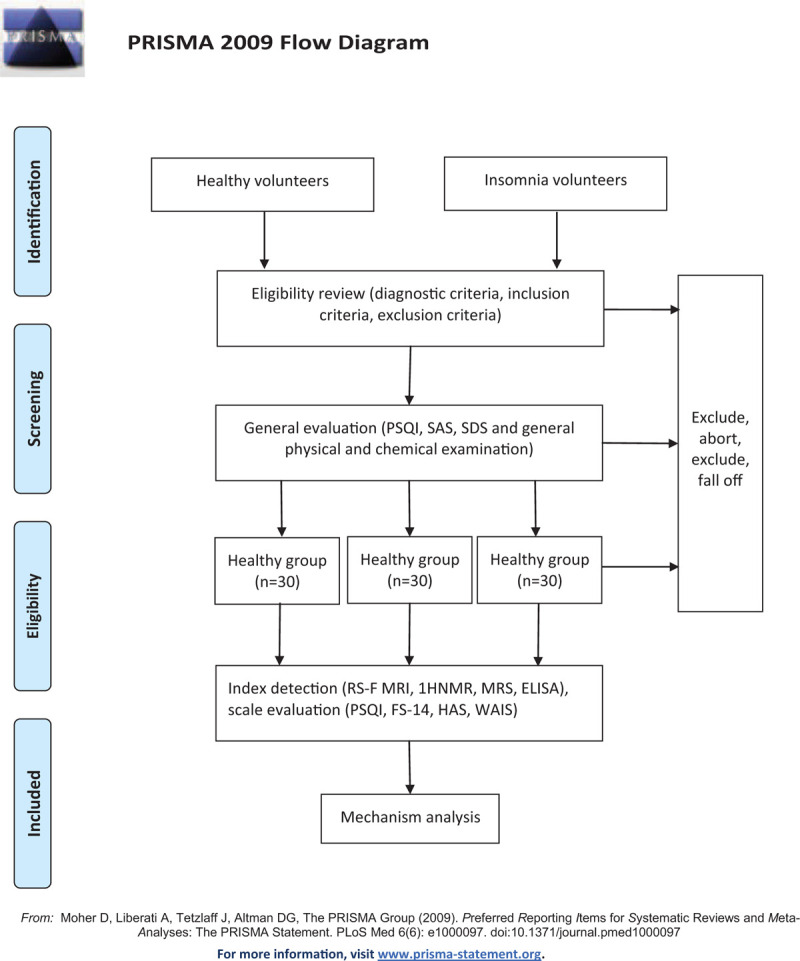
Trial flow chart.

The patients must properly understand and sign informed consent. Pittsburgh Sleep Index scale (PSQI), Hyperarousal scale (HAS), Hamilton Depression Scale (HAMD), and HAMA scores will be used to potentially determine whether participants meet the inclusion criteria. Patients who met the criteria will thereafter be included in the study (Fig. 1).

### 2.4 Ethical issues

This research project has been reviewed and approved by Ethics Committee of Affiliated Hospital of Changchun University of Traditional Chinese Medicine (CCZYFYLL2021 Standard word -016). This research project was funded by the National Natural Science Foundation of China (project approval Number: 82074569).

### 2.5 Diagnostic criteria

#### 2.5.1 Diagnosis of insomnia: refer to “The guidelines for the diagnosis and treatment of insomnia in Chinese Adults” (2017 edition)

The patient will have to display one of the following symptoms of insomnia without obvious inducement, namely decline in the sleep quality, difficulty falling asleep, persistent disorder, early awakening, or going back to sleep after waking up without completely recovering. Those patients who still have the above symptoms when they meet the sleeping conditions in a suitable environment will be considered. The major complaint will be at least one of the following sleep-related functional impairments. Fatigue or general discomfort; impaired concentration, maintenance of attention, or memory; decrease in ability to study, work and/or socialize; emotional irritability or irritability; day dreaming; behavioral problems (e.g., hyperactivity, impulsivity, interest, loss of energy; prone to errors and accidents during work or driving; tension, headache, dizziness, or other symptoms related to lack of sleep and excessive attention to sleep or dissatisfaction with the quality of sleep).

#### 2.5.2 Diagnosis of insomnia caused by deficiency of heart and spleen: refers to TCM clinical practice guidelines for insomnia (WHO/WPO); and diagnostic criteria for insomnia of “Deficiency of heart and Spleen syndrome” in guiding principles of clinical research on new Chinese medicine (Trial)

The main symptoms that will be noted will be: excessive dreams, patients could easily wake and thereafter experienced difficulties to fall asleep again, palpitations forgetfulness. The major syndromes which will be noted include that of tired appearance, weak limbs unhealthy complexion, dizziness, poor appetite, thin tongue moss, and a weak pulse.

### 2.6 Inclusion criteria

#### 2.6.1 Inclusion criteria for chronic insomnia (heart and spleen deficiency type)

The participants will have to meet the diagnostic criteria of chronic insomnia and the TCM symptoms of heart and spleen deficiency, and fulfill the following conditions: they will have to meet the diagnostic criteria for chronic insomnia, and meet the heart and spleen deficiency related symptoms of TCM. They must be between 50 and 55 years of age. They must not have been enrolled in any other clinical studies. They will have to sign informed consent and voluntarily participate in this research. Their Pittsburgh Sleep Index (PSQI) index score must be >7, the points of the Athens (AIS) scale score must be >6. The standard score of Depression Scale (SDS) and Anxiety Scale (SAS) must be ≤60. They must not experience work and life pressure during the observation period. They must not suffer from major cardiovascular and cerebrovascular diseases and other chronic diseases; their blood and urine analysis must be normal, as well as electrocardiogram, electroencephalogram, liver and kidney function test, blood sugar, and blood lipids and other inspection indicators must fall in the normal range. Patients with the above conditions lasting >1 year and frequency ≥3 times/wk will be included in this study.

#### 2.6.2 Inclusion criteria for healthy volunteers

Age 50–55, has been living in Changchun area, right-handed, moderate body size. They must have no religious belief, no tobacco, alcohol, coffee, tea, and other bad habits. They must be married with a moderate income. They must not be suffering from excessive pressure in work and life during the observation period. They must be in good health, and must not suffer from cerebrovascular disease and other chronic diseases. The woman must not be pregnant, menstruating, or lactating. The routine physical and chemical examinations such as ECG, hematuria routine, liver and kidney function, and other indicators must be normal. They must not display symptoms of insomnia, with PSQI, SAS, and SDS scores will have to be assessed as normal.

### 2.7 Exclusion criteria

However, if the subject meets any of the below indicated criteria, they will not be able to participate in this trial.

Age is not in the range of 50–55 years. PSQI index is ≤7. The AIS index must be ≤6. Insomnia is being caused by excessive anxiety, depression, etc, SDS is >60 points or SAS is >60 points. They may be suffering from breathing-related sleep disorders, circadian rhythm sleep disorders, and sleep dysfunction. Patients suffering from insomnia caused by pain, fever, cough, and other diseases. Patients with severe primary diseases with internal organs and hematopoietic system. Patients with mental illness. Patients with skin infections or skin allergies at the treatment site. Progressive malignant tumors or other serious wasting diseases. Those who may have poor compliance and will not be able to treat as prescribed. Those who may be unable to judge the efficacy or incomplete data. Patients with insomnia caused by drug abuse or drug treatment. Insomnia caused by long-term excessive drinking, coffee, strong tea, and other unhealthy lifestyle related factors.

### 2.8 Termination of the standard

After enrollment, the trial will be effectively terminated if one of the following symptoms occurs.

The patients suffer from the severe adverse reactions that may occur during the course of the study (medical certificate issued by the observing doctor); severe complications or deterioration of the subjects during the study period; the subjects request to willingly withdraw from the clinical study halfway; patients who may be uncooperative and have poor compliance. Additionally, if the conditions for the termination of the test must be met after being processed by the competent doctor in accordance with the standardized working procedure (SOP), the test shall be terminated immediately. The researchers will keep a detailed record of the reasons and time of withdrawal of the participants from the study. Patients who exceed at least 1/2 course of treatment will be able to enter the efficacy statistics and must properly maintain a random medical record number.

### 2.9 Stripping and shedding criteria

After enrollment, patients who meet one of the following criteria will be terminated from the trial.

Exclusion criteria: Those who might fail to meet the inclusion criteria and have entered the study; those who might to follow the prescribed treatment or incomplete data affecting the efficacy evaluation and safety evaluation; those who might have used the different therapeutic methods prohibited by this protocol; and those who might change the treatment plan midway by themselves.

Shedding criteria: The subjects with poor compliance and self-withdrawal during the course of treatment; those who might display serious adverse events or in those in which complications might occur; and those who will be unable to continue the treatment for various reasons.

### 2.10 Treatment programs

After being enrolled, 30 patients will be treated with abdominal vibration and ring kneading therapy. The main techniques that will be used include rubbing the abdomen, shaking the abdomen, and pressing the abdomen. The main points to be considered will be Zhongwan (CV12), Shenque (CV8), Zhongji (CV3). The acupoint location that will be used refers to the 2006 National Standard of the People's Republic of China (GB/T12346-2006) “Acupoint Name and Location.”

During the treatment, room temperature will be kept appropriate, and the environment quiet and tidy. The patient will be laid in the supine position, exposing the abdomen and maintain it in a relaxed state. The doctor will sit on the right side of the patient. The doctor will rub the entire abdomen clockwise with CV 8 as the center for 5 minutes, up to CV 12, and down to CV 3. The left and right sides will be placed between the left and right meridians of the foot Taiyin Spleen meridian, so as to regulate the air movement and allow the blood to flow. Thereafter, the whole abdomen will be kneaded with both hands for 10 minutes to adjust the internal organs and to balance the yin and yang. CV 12 will be vibrated with one hand for 2 minutes, palm CV 8 for 3 minutes, and flash CV 3 for 5 minutes with one hand, at a frequency of >200 times per minute to invigorate the spleen and stomach, and replenish qi and blood. Thereafter, abdomen will be pressed with the palm of the hand centered on CV 8, it will be pressed deeply while breathing in, and will be lifted gently while breathing out, for 5 minutes, to induce the air to return to the original body, and to calm the mind. Patients will undergo the therapy for 30 minutes duration each time, once a day, 5 days as a course of treatment, the treatment interval will be 2 days, and intervention will be conducted over 4 courses. There will be a follow up conducted once during the 4th week after the treatment.

### 2.11 Evaluation indicators

#### 2.11.1 Treatment rating scale

Based on the scale evaluation, PSQI, FS-14, HAS, and WAIS scales will be used to evaluate heart rate variability (HRV) of patients in each group before the first treatment, after the last treatment, and 4 weeks after the end of treatment (follow-up).

#### 2.11.2 Rs_fMRI

Sleeping patients will be subjected to Rs_fMRI in the morning of the day before the start of treatment and the morning after the last treatment after completion of the 4 courses of treatment. Siemens will be first used to perform conventional MRI structural images of the brain, namely T1 and T2 scans. The test will be terminated if the brain structure was abnormal. When the standard Rs_fMRI test will be performed, the patients will be required to lie down, be quiet, close their eyes, and try not to think when scanning is being conducted. The three-dimensional “∗.DCM” file will be obtained after scanning as per the parameters design of Siemens medical system, and the original data will be saved. The image will be preprocessed by DPARSF software including conversion of “.dcm ”to“.nfTI,” Slice Timing, head Timing (Realign), Normalize, Smooth etc. The pretreatment parameter TR will be set at 2 mm; the first 10 time points will be removed; FALFF Band (Hz): 0.01 to 0.1; ReHo Ouster: 27 Voxels. Voxel Slice [3,3,3], others with default values. SPM12 software will be used for statistical analysis of the images, and Xjview software will be used for image analysis.

#### 2.11.3 MRS detection

After the Rs_fMRI detection, imaging will be continued on the Siemens 3.0T scanner. The parameters will be set as SVS-SE-135 MRSI. The collection method used will be stimulated echo (STEAM). TR/TE = 2000/30 ms. Voxel size will be 20 × 20 × 20 mm^3^; the bandwidth (bandwidth) used will be 1200 Hz; acquisition duration will be 853 ms. The pixels will be obtained at 148%. The cephalopods (HFS) will be taken from the patient and the scanning location will be the hypothalamus region of the head. The results will be saved in the format of “.jpg.” The displacement value (PPM) of GABA and 5-HT will be determined and measured. The contents of GABA and 5-HT in hypothalamus will thereafter observe.

#### 2.11.4 ^1^HNMR test

The blood samples will be obtained from an empty stomach in the morning before the beginning of admission treatment in the healthy group and the patient group, and on the following morning after the end of treatment. Before blood collection, the patient will be required to rest for 30 minutes, venous blood will be extracted, the serum will be centrifuged and stored in a refrigerator at –80 °C. Thereafter, before the detection, the serum samples will be resuscitated at the room temperature, and 400 L samples will be extracted and placed in a centrifuge tube with 30 L phosphate buffer (K2HPO4/NaH2PO4, 1.5 mol/L, pH = 7.4). Then, 170 L heavy water will be added and mixed. The serum samples will be centrifuged at 12,000r/min at 4 °C for 10 minutes, and 500 L supernatant will be taken and placed in a 5 mm NMR tube. The various common metabolites, differential metabolites, potential metabolic biomarkers, and metabolic pathways will be analyzed among each group. Superconducting NUCLEAR magnetic resonance spectrometer will be used for the data collection, and MestReNova-12.0.3-21384 software will be used for original data processing. Principal component analysis and orthogonal partial least squares analysis in multivariate statistical analysis will be performed using SIMCA-P14.0 software. MetabaAnlyst4.0 database will be employed and online metabolic data, http://www.metaboanalyst.ca, will be used to complete the metabolic pathway analysis.

#### 2.11.5 ELISA detection

The estimation of the serum CRH, CRHR1, ACTH, COR, GABA, NE, PGE2, 5-HT levels will be carried out using enzyme linked immunosorbent assay (ELISA). The serum samples will be prepared and processed according to the instructions of each ELISA kit to calculate the OD value and to calculate the content using the curve formula.

### 2.12 Quality control

The study quality management will be strictly carried out with reference to the Quality Management Standard for Drug Clinical Trials. The Massage therapy was in the charge of special personnel. A thorough training, practice, and assessment of the manipulation operation will be conducted before the start of the treatment to ensure that the time, intensity, and frequency of manipulation operations will be relatively consistent and will meet clinical standardization requirements as far as possible.

### 2.13 Statistical method

Spss23.0 statistical software will be used for statistical analysis for all the data generated. Chi-square test will be used for enumeration data, paired *t* test will be used for the comparison of measurement data before and after the treatment. One-way analysis of variance (ANOVA) will be used for comparison between the treatment groups, with *P* < .05 as difference and *P* < .01 as significant difference.

## 3 Discussion

The treatment method of this project is derived from the Affiliated Hospital of Changchun University of Traditional Chinese Medicine, which regulates zang-fu organs through affecting different meridians. According to the principle of “meridian adjustment viscera,” the idea of “improve the brain by treating the intestines” has been put forward. Abdominal massage treatment of insomnia has been carried out for 15 years. It has been found that good clinical effects have been achieved, but there is still a lack of basic research. This project is an important example of the beneficial impact of abdominal massage in the treatment of neurological diseases, which could possibly explain the treatment of brain diseases through abdomen massage, and also explain to some extent the theory proposed in Huangdi Neijing that “if you feel uncomfortable in your stomach, you will not sleep well.” The hypothesis of the study was that abdominal massage can treat insomnia through affecting HPA axis, hence a scientific and systematic research and analysis on this clinical practice was carried out, the mechanism of action of abdominal massage in treating insomnia was analyzed, and the findings could potentially provide novel ideas for the future clinical practice and scientific research.

The brain–gut interaction mechanism refers to the relationship between the gastrointestinal tract and the brain. A number of the studies have established that the signal regulation system of the gastrointestinal tract and the brain is the only system in the body jointly controlled by CNS, ENS, and ANS.^[12]^ Moreover, sympathetic neurons and parasympathetic neurons and the adrenal medulla and adrenal cortical hormone from the brain to the intestinal descending pathways, and nerve–endocrine–immune network and information transmission can constitute the intestinal flora from the gut to the brain uplink channel, in the interactive process mediated by the hypothalamus-pituitary-adrenal (HPA) axis whose major function is to coordinate the important mechanism of the human nervous system and endocrine system, not only to the nerve center of stress reaction, but also to participate in regulation of the gastrointestinal smooth muscle contraction and mucous membrane of transshipment.^[13]^ The gastrointestinal motility function, gastrointestinal secretion function, and intestinal permeability change could have a significant impact on the gastrointestinal microflora and thereby modulate the physiological stability of its metabolism. The intestinal microbial metabolism not only interacts with the intestinal nervous system, but also can directly interact with the central nervous system.

Based on the theory of brain–gut interaction in the treatment of insomnia with abdominal massage, this study will further analyze the possible relationship between hypothalamus and brain–gut axis, CRH/CRHR1 pathway and sleep, and between HPA axis and sleep. On the basis of previous research and literature analysis, it has been established that the hypothalamus CRH/CRHR1 signaling pathway may play a pivotal role in the initiation of the HPA axis, coordination of the important pathways in the human nervous and endocrine systems but can also participate in the central nervous stress reaction, and can be involved in regulating the gastrointestinal smooth muscle contraction and mucous membrane of transshipment and thereby may play an important role in the process of brain gut interaction. Therefore, the proposed hypothesis that the treatment of insomnia by abdominal vibration and ring massage could be related to the intervention of hypothalamic CRH/CRHR1 signaling pathway, activation of HPA axis, regulation of different mediators such as ACTH, COR, 5-HT, GABA, and other metabolites in the body, thus contributing to the maintenance of neuroendocrine homeostatic state will be analyzed.

In this project, insomnia patients will be used as research objects, and scale screening, fMRI test, MRS test, serum ^1^HNMR test, and serum ELISA test will be carried out, so as to clarify the efficacy and influence on the HPA axis as a result of abdominal vibration and ring massage on insomnia patients from the clinical perspective.

CRH is the promoter of the HPA axis. CRH not only regulates the activity of the NE and 5-HT systems in the locus coeruleus, but also that of ACTS on the CRH receptors in the hypothalamus.^[10]^ There are 2 different kinds of CRH receptor reporter in mammals, first one is a type I adrenocorticotropic hormone releasing hormone receptor (CRHR1), and the other is a type II adrenocorticotropic hormone releasing hormone receptor (CRHR2). The affinity of CRH to CRHR1 is higher than that of CRHR2. After CRH neurons in the hypothalamic paraventricular nucleus (PVN) are stimulated by stress, they will synthesize and release CRH and transport it to the anterior pituitary area, where it will bind to CRHR1 receptors on the cell membrane surface, and activate G-protein and downstream pathways after the reaction occurs, to stimulate the pituitary gland to produce and secrete ACTH. ACTH reaches the adrenal glands through peripheral blood circulation, and causes the release of corticosterone, cortisol, and other substances by ACTH.^[14]^ It has been previously reported that CRH/CRHR1 pathway in the hypothalamus can regulate sleep related mechanism(s), but evidence to support the results are not consistent, and findings are more focused on the downstream pathways associated with metabolism such as detection of ACTH, cortisol, etc. However, there is no exact experimental data to indicate that how patients with insomnia could exhibit stress response in the hypothalamus. CRH is the CNS is one of the main stress factor, there is no exact experimental data for patients with insomnia exact stress changes in the hypothalamus. At present, there are few studies related to the role of CRH on insomnia, and the experimental results are not consistent. This research program can hopefully prove that the vibrating belly and ring kneading method can treat insomnia through modulating the CRH/CRHR1 pathway of brain–gut interaction, and thereby verify the correctness of the brain–gut interaction theory.

### 3.1 Trial status

On April 8, 2021, the medical Ethics Committee of Affiliated Hospital of Changchun University of Traditional Chinese Medicine approved the study plan (CCZYFYLL2021 Standard word -016). Due to the delay in recruitment and preparation time, the trial run will begin in June 2021, and we expect the recruitment of the volunteers to be completed by June 2022.

## Author contributions

**Conceptualization:** Cong de Yu, Hongshi Zhang.

**Funding acquisition:** Hongshi Zhang.

**Investigation:** Peng Liu, Congcong Shi, Jiajun Zhao.

**Methodology:** Zhang Ye, Hongshi Zhang.

**Project administration:** Zhang Ye, XiaoYu Zhi.

**Resources:** Zhang Ye, Hongshi Zhang.

**Writing – original draft:** Hongshi Zhang.

**Writing – review & editing:** Zhang Ye.
